# Elevated Expression of miR-210 Predicts Poor Survival of Cancer Patients: A Systematic Review and Meta-Analysis

**DOI:** 10.1371/journal.pone.0089223

**Published:** 2014-02-20

**Authors:** Jian Wang, Jiqing Zhao, Mengjing Shi, Yu Ding, Huiqin Sun, Fahuan Yuan, Zhongmin Zou

**Affiliations:** 1 Institute of Toxicology, School of Preventive Medicine, Third Military Medical University, Chongqing, China; 2 Department of nephrology, Xinqiao Hospital, Chongqing, China; Columbia University, United States of America

## Abstract

**Background:**

MiRNAs are important regulators of different biological processes, including tumorigenesis. MiR-210 is a potential prognostic factor for survival in patients with cancer according to previous clinical researches. We conducted a systematic review and meta-analysis to summarize the significance of increased miR-210 expression in the prognosis of indicated cancers.

**Methodology/Principal Findings:**

The present systematic review and meta-analysis of 16 researches included 1809 patients with 7 different types of cancers from 7 countries, and aimed to explore the association between miR-210 expression and the survival of cancer patients. Over-expression of miR-210 may predict poor overall survival (OS, HR = 1.33, 95% CI: 0.85–2.09, *P* = 0.210), but the effect was not significant. While the predictive effect on disease-free survival (DFS, HR = 1.89, 95% CI: 1.30–2.74, *P* = 0.001), progression-free survival (PFS, HR = 1.20, 95% CI: 1.05–1.38, *P* = 0.007) and relapse-free survival(RFS, HR = 4.42, 95% CI: 2.14–9.15, *P* = 0.000) for patients with breast cancer, primary head and neck squamous cell carcinoma (HNSCC), renal cancer, soft-tissue sarcoma, pediatric osteosarcoma, bladder cancer or glioblastoma was certain. Subgroup analysis showed the limited predictive effect of over-expressed miR-210 on breast cancer OS (HR = 1.63, 95% CI: 0.47–5.67, *P* = 0.443), breast cancer DFS (HR = 2.03, 95% CI: 0.90–4.57, *P* = 0.088), sarcoma OS (HR = 1.24, 95% CI: 0.20–7.89, *P* = 0.818) and renal cancer OS (HR = 1.16, 95% CI: 0.27–4.94, *P* = 0.842).

**Conclusions/Significance:**

This systematic review and meta-analysis suggests that miR-210 has a predictive effect on survival of patients with studied cancer types as indexed by disease-free survival, progression-free survival and relapse-free survival. While the predictive effect on overall survival, breast cancer overall survival, breast cancer disease-free survival, sarcoma overall survival and renal cancer overall survival was not statistically significant.

## Introduction

MicroRNAs are a class of small non-coding RNAs approximately 21 nucleotides in length, which regulate about 30% of human genes at post-transcription level and subsequently affect the biological processes of cells. They have been identified to repress the translations and/or promote the degradations of their target mRNAs by specifically binding to the 3′ untranslated regions of the mRNAs [1]. Plenty of works have confirmed the significant role of miRNAs in regulating cell proliferation, differentiation, apoptosis, metabolism, development and immunity since their first discovery in 1993 [2].

In 2002, a research depicted a decreased expression of miRNA-15a and miRNA-16-1 in B-cell chronic lymphocytic leukemia, which was the first evidence of an association between miRNA and cancer [3]. After that, different groups described the expression profiles of miRNA in various types of cancers [4]. Now it’s widely accepted that miRNAs function as cancer suppressors or oncogenes.

MiR-210 is a well-known hypoxia-inducible miRNA that expressed in a wide range of cells. It’s a key factor involving in cell proliferation, mitochondrial respiration, DNA repair, vascular biology and angiogenesis [5]. Since 2007 [6], [7], a host of works have showed the prognosis effect of miR-210 on different cancers, such as breast cancer [8], epithelial ovarian cancer [9], diffuse large B-cell lymphoma [10], lung cancers [11], pancreatic adenocarcinomas [12], kidney cancers [13] and so on. Most of the researches revealed an elevated expression of miR-210 in cancer tissues compared with normal tissues, and which is related to a poor survival outcome [8], [11], [14], [15], [16], [17], [18], [19], [20], [21]. However, there were still reports showing the insignificant or opposite results [8], [9], [10], [14], [22], [23], [24]. Therefore, it is essentially necessary to carry out a systematic review and meta-analysis to summarize the published global findings, and get a better understanding on the significance of miR-210 expression in the prognosis of cancer patients.

In the current study, global related literatures were collected to conduct a systematic review and meta-analysis, and the risk of increased miR-210 expression to the survival of cancer patients was successfully assessed.

## Materials and Methods

This meta-analysis was conducted totally following the guidelines of Preferred Reporting Items for Systematic Reviews and Meta-Analyses (PRISMA) 2009 Checklist (http://www.prisma-statement.org/statement.htm) and Meta-analysis of Observational Studies in Epidemiology group (MOOSE) [25]. The PRISMA 2009 Checklist and MOOSE Checklist for our study are shown in supplementary materials (Checklist S1 and Checklist S2).

### Identification of Eligible Studies

We systematically and carefully searched the online Pubmed (http://www.ncbi.nlm.nih.gov/pubmed) and Embase (http://www.embase.com/home) from 1993 to December 4th, 2013 to collect related literatures, using “miR-210 and cancer”, “miR-210 and carcinoma”, “miR-210 and tumor”, “miR-210 and neoplasm” as keywords respectively. We set no advanced limitations when searching the both databases. All the searching results were checked by going through the titles and abstracts. The duplications were removed directly. Records were eligible if they met the following criteria: (i) they studied patients with any type of carcinoma; (ii) they measured the expression of miR-210 in cancer tissue or serum; and (iii) they investigated the relationship between miR-210 expression level and survival outcome.

Articles were excluded according to the following criteria: (i) reviews, comments, conference abstracts, letters or laboratory studies, (ii) with only the expression of a set of miRNAs other than a separated one, (iii) lack of key information or cannot estimate HR by the shown data.

In case of multiple reports on the same trial, we selected the one with more details for meta-analysis. The references of the selected reports were also checked for any possible eligible studies. As to articles without essential data, we sent request to the authors by E-mail, and only qualified the one with enough provided data.

Information of the eligible reports, such as titles, abstracts and full texts was independently and carefully identified by three reviewers (Wang, Shi and Ding). Zhao and Sun checked these extracted articles for a second time. These reviewers (Wang, Shi, Ding, Zhao and Sun) discussed to resolve any disagreement or consulted with senior reviewers (Zou and Yuan).

### Quality Assessment

All the included studies were evaluated according to the critical review checklist of the Dutch Cochrane Centre proposed by MOOSE [25]. The key points were as following: (i) enough information of study population, (ii) enough information of the carcinoma, (iii) clear description of study design, (iv) clear description of outcome assessment, (v) enough description of miR-210 measurement, (vi) clear description of cut-off of miR-210 and (vii) sufficient period of follow-up. We excluded the studies without mentioning all these seven points.

### Data Extraction and Conversion

Data were extracted independently in standardized data-collection forms. The extracted data included the following details: (i) publication information: first author’s last name and publication year; (ii) patients’ characteristics: sample size, disease, stage of disease, histological type and follow-up; (iii) miR-210 measurement and cut-off value; and (iv) HR of elevated miR-210 for overall survival (OS), recurrence-free survival (RFS), progress-free survival (PFS), disease-free survival (DFS), as well as their 95% confidence intervals (CI) and *P* values. If available, the HRs with their 95% CIs and *P* values were collected from the original article or the corresponding E-mails. If not, we calculated HRs and their 95% CIs using the data of observed deaths/cancer recurrences, the data of samples in each group or the data provided by the authors. If only Kaplan–Meier curves were available, we extracted data from the graphical survival plots and estimated the HRs. All the calculations mentioned above were based on the methods provided by Parmar, M. K. et al [26] and Tierney, J. F. et al [27].

### Statistical Analysis

The test of heterogeneity of combined HRs was carried out using Cochran’s Q test and Higgins I-squared statistic. A *P* value of <0.05 and/or I^2^>50% was considered statistically significant. A random effect model (Der Simonian and Laird method) was applied if heterogeneity was observed, while a fixed effect model was used in the absence of between-study heterogeneity (*P*≥0.05, I^2^≤50%). The factors contributed to heterogeneities were analyzed by sub-group analysis, meta regression or sensitive analysis. Publication bias was evaluated using the funnel plot with the Egger’s bias indicator test [28]. All analyses were performed using ‘STATA: Data Analysis and Statistical Software’ V11.

## Results

### Study Characteristics

**Figure 1:**
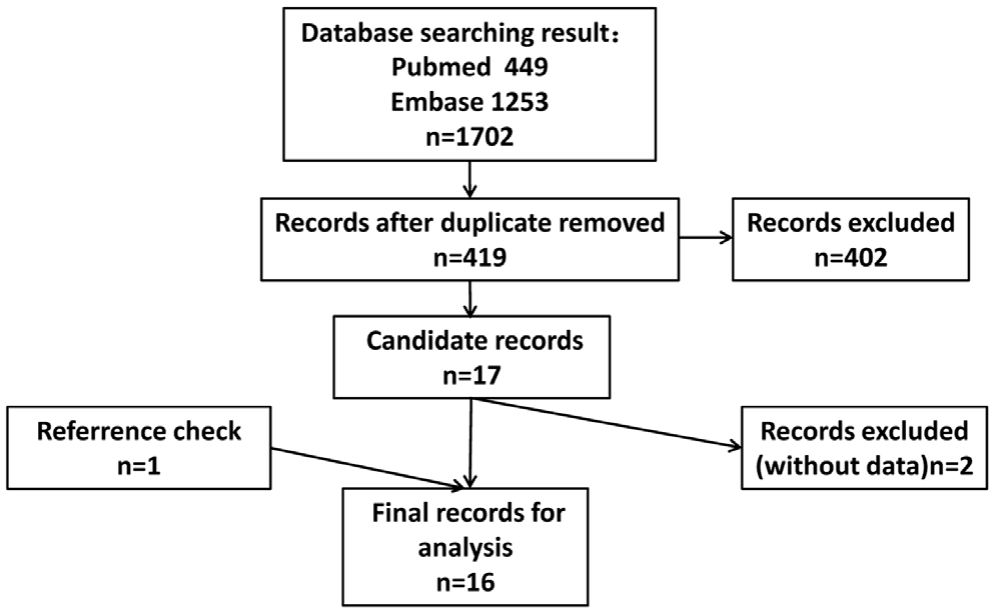
The flowchart showed the selection of studies for meta-analysis.

We collected 449 records from Pubmed and 1253 from Embase in the primary search, and excluded 1283 duplicates from the initial 1702 records. After screening the titles, abstracts, publication types and full texts of the rest 419 records, 17 records were qualified for the present study. Then, the references of these qualified records were manually checked and 1 additional record [29] was found and included. Two articles showing only the conclusion without essential data were excluded. Finally, we got 16 records for systematic review and meta-analysis [8], [14], [17], [19], [20], [22], [23], [24], [29], [30], [31], [32], [33], [34], [35], [36]. Fig. 1 showed the flow diagram of candidate study selection in our study.

The main information of the 16 articles was summarized in Table 1. All of the 16 articles were retrospective in design. The collected 1809 patients were from the United Kingdom, Germany, Australia, Greece, Japan, China and the United States. The patients were diagnosed with a variety of cancers, including breast cancer, primary head and neck squamous cell carcinoma (HNSCC), renal cancer, soft-tissue sarcoma, pediatric osteosarcoma, bladder cancer and glioblastoma. Of all the studies, 7 were focused on breast cancer (n = 955) [8], [17], [19], [29], [32], [33], [36], 3 on renal cancer (n = 182) [22], [31], [35] and 2 on sarcoma (n = 546) [23], [34]. Fourteen of the 16 studies assessed miR-210 expression by quantitative PCR, a widely used method for miRNA quantitation. All studies measured miR-210 expression in collected tumor samples except one research in serum [33]. Even most researches preferred mean/media as cut-off values, there were still records using tercentiles [19], [23], quartile [8], [14], [33] or maximum normal tissue expression value [31] instead. The follow-up time ranged from 20 months to 180 months, and reached more than 100 months in 11 researches. All the HRs and their 95% CIs in the collected articles were shown in Table 2. We carefully summarized all the available assay details in Table 3. Firstly, except 2 researches mentioned nothing about their internal references, 7 researches used 1 molecular as their internal references, 3 researches used 2 and 4 researches used 3. Secondly, 6 researches were lack of the information of expression analysis method, 7 researches used ΔΔCt, 1 used Cp value, 1 used Cq value, and 1 used ΔΔCq value. Thirdly, the expressions of miR-210 were quite different from each other. Two researches did not supply the expression levels of miR-210, 1 research only listed the *P* value, 7 researches used fold changes as the expression level of miR-210, and the rest 6 researches showed the miR-210 expression level normalized to their internal references. As to the risk evaluation methods, 2 researches used univariate regression, 10 researches used Kaplan-Meier curves and the rest 4 used Multiple Cox proportional harzard model.

**Table 1:** Summary table of meta-analysis.

	origin ofpopulation	studydesign	diseases	N =	Stage	miR-210assay	cut-off	survivalanalysis	Hazard ratios	follow-upmonths
Cai, 2013	China	R	pediatric osteosarcoma	92	–	qRT-PCR	median	OS, PFS	reported	82 (10–133)
Camps, 2008	UK	R	breast cancer	219	I–III	qRT-PCR	median/quartiles	OS, DFS	reported	120
Gee, 2009	UK	R	primary HNSCC	46	I–IV	qRT-PCR	median/quartiles	OS, RFS	AP/DE/SC	clinical 41(1–53);DFS 40(2–53)
Greither,2009	Germany	R	PDAC	56	–	qRT-PCR	median	OS, DFS	reported/DE	15.99(1–61)
Greither,2012	Germany	R	soft-tissue sarcoma	78	I–IV	qRT-PCR	tercentiles	DSS	AP	120
Madhavan, 2012	Germany	R	metastatic breast cancer	269	–	qRT-PCR	lower quartile	OS, PFS	SC	20
Markou,2013	Greece	R	breast cancer	112	I–III	qRT-PCR	median	OS,DFS	reported	148.8
McCormick, 2012	UK	R	renal cancer	40	T1–T3	qRT-PCR	median	OS	SC	120
Neal, 2010	Australia	R	renal cancer	31	pT1–pT4	qRT-PCR	maximum normal tissueexpression value	OS	SC	140
Qiu, 2013	China	R	glioblastoma	458	–	qRT-PCR	median	OS, PFS	SC	130
Radojicic, 2011	Greece	R	breast cancer	49	–	qRT-PCR	mean	OS, DFS	AP/DE/SC	116
Rothe, 2011	UK	R	breast cancer	73	I–III	qRT-PCR	median	RFS	reported	120
Toyama, 2012	Japan	R	triple-negative breast cancer	40	I–III	qRT-PCR	tercentiles	DFS*	reported	168
Volinia, 2012	USA	R	breast cancer	58	–	qRT-PCR	median	OS/DFS	SC	180
Wotschofsky,2012	Germany	R	renal cell carcinoma	111	T1–T4	qRT-PCR	median	OS	reported	8.0 (3.4–44.8)
Zaravinos, 2012	Greece	R	bladder cancer	77	–	qRT-PCR	median	OS	reported	50

Study design is described as consecutive patients (C), prospective (P) or retrospective (R). –, not reported; HNSCC, head and neck squamous cell carcinomas; PDAC, pancreatic ductal adenocarcinoma; qRT-PCR, quantitative real-time PCR; OS, overall survival; DSS, disease-specific survival; DFS, disease-free survival; RFS, relapse-free survival; PFS, Progression-free survival; AP, author provided; DE, data-extrapolated; SC, survival curve.

*the OS data is obviously not consistent with the survival curves, so only provided DFS is used for analysis.

**Table 2:** Summary table of HRs and their 95% CI.

study	year	Disease	HR	95% CI and P value	outcome
Madhavan	2012	metastatic breast Cancer	0.31	0.09–1.02, P = 0.00023	OS
Zaravinos	2012	bladder cancer	0.34	0.11–1, P = 0.049	OS
Wotschofsky	2012	renal cell carcinoma	0.39	0.12–1.23, P = 0.109	OS
Geither	2012	soft-tissue sarcoma	0.5	0.23–1.12, P = 0.084	OS
Markou	2013	breast cancer	1.028	0.486–2.174, P = 0.943	OS
Markou	2013	breast cancer	1.049	0.581–1.895, P = 0.873	DFS
Neal	2010	renal cancer	1.15	0.15–8.65, P = 0.189	OS
Madhavan	2012	metastatic breast Cancer	1.18	0.84–1.67, P = 0.107	PFS
Qiu	2013	Glioblastoma	1.19	1.02–1.37, P = 0.0212	PFS
Qiu	2013	Glioblastoma	1.33	1.13–1.57, P = 0.0077	OS
Volinia	2012	breast cancer	1.41	0.32–6.16, P = 0.013	DFS
Gee	2009	primary HNSCC	1.49	0.22–10.09, P = 0.008	OS
Volinia	2012	breast cancer	1.57	0.38–6.52, P = 0.006	OS
Gee	2009	primary HNSCC	1.58	0.12–19.93, P = 0.003	DFS
Radojicic	2011	breast cancer	2	0.29–13.75, P = 0.1220	OS
Geither	2009	PDAC	2.48	1.32–4.68, P = 0.005	OS
Geither	2009	PDAC	2.5	1.24–5.02, P = 0.01	DFS
Cai	2013	pediatric osteosarcoma	2.6	0.8–7.2, P = 0.02	PFS
McCormick	2012	renal cancer	3.01	1.39–6.51, P = 0.005	OS
Cai	2013	pediatric osteosarcoma	3.3	1–8.2, P = 0.01	OS
Radojicic	2011	breast cancer	3.72	0.75–18.75, P = 0.0658	DFS
Camps	2008	breast cancer	4.07	1.7–9.75, P = 0.002	DFS
Toyama	2012	Triple-negative Breast cancer	4.39	1–19.28, P = 0.036	RFS
Rothe	2011	breast cancer	4.43	1.91–10.16, P = 0.0005	RFS
Camps	2008	breast cancer	11.38	4.1–31.65, P<0.001	OS

**Table 3:** Summary table of the miRNA detection and HR calculation.

study	year	internal reference	miR-210 expression	risk evaluation method
Madhavan	2012	cel-miR-39	5.17, P<0.05	Kaplan-Meier
Zaravinos	2012	RNU1A1, RNU5A, RNU6B	∼1.3, P = 0.4554	Univariate regression
Wotschofsky	2012	miR-28, miR-103, miR-106a	∼0.63, P = 0.0.193	Univariate regression
Geither	2012	U18	127.851–870.550	Mul Cox proportional harzard model
Markou	2013	miR-191	–, P = 0.708	Kaplan-Meier
Neal	2010	RNU43, RNU48	8, P = 0.05	Kaplan-Meier
Qiu	2013	–	–	Kaplan-Meier
Volinia	2012	–	–	Kaplan-Meier
Gee	2009	RNU43, RNU44, RNU48	2.02(0.15–8.18), P>0.05	Kaplan-Meier
Geither	2009	18S rRNA	0.31 (0.006–122.9)	Mul Cox proportional harzard model
Cai	2013	RNU6B	∼1.15, P<0.001	Kaplan-Meier
McCormick	2012	RNU44, RNU48, RNU6B	10, P<0.001	Kaplan-Meier
Radojicic	2011	RNU5A, RNU6B	3.74±4.01, P<0.001	Kaplan-Meier
Camps	2008	RNU43	5.48(0.08–67.81), P<0.05	Mul Cox proportional harzard model
Toyama	2012	RNU6B	11.1±2.60, P<0.001	Mul Cox proportional harzard model
Rothe	2011	RNU44, RNU48	∼3.43, P = 0.009	Kaplan-Meier

### Meta-analysis Results

**Figure 2:**
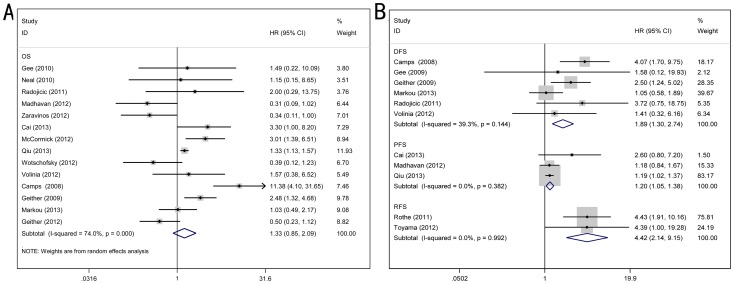
Forrest plotsof studies evaluating hazard ratios of high miR-210 expression. (A) Overall survival test. The survival data from 14 records were pooled to calculate overall survival. The random effects analysis model showed the pooled HR for overall survival is 1.33 with 95% CI 0.85–2.09, and *P* value is 0.210. (B) Survival data were presented as disease-free survival, relapse-free survival and progression-free survival. The fix effect analysis model was used to calculate the pooled HRs, and the results were HR = 1.89 (95%CI: 1.30–2.74, *P* = 0.001) for DFS, HR = 4.42 (95%CI: 2.14–9.15, *P* = 0.000) for RFS, and HR = 1.20 (95%CI: 1.05–1.38, *P* = 0.007) for PFS.

For studies evaluating OS of patients, a pooled HR and its 95% CI were calculated with a random model because of the high heterogeneity between studies (*P*  = 0.000, I^2^ = 74.0%). The result showed that higher expression of miR-210 may predict poor OS, and the pooled HR was 1.33 (95% CI: 0.85–2.09) (Fig. 2a), however, the effect did not reach the level of statistical significance (*P* = 0.210). As the studies testing DFS were not of obvious heterogeneity (*P*  = 0.144, I^2^ = 39.3%), we used a fixed model to pool the HRs, and the pooled HR 1.89 (95% CI: 1.30–2.74) (Fig. 2b) revealed that over-expression of miR-210 significantly predicted poor DFS (*P* = 0.001). For articles reporting PFS (n = 3) and RFS (n = 2) as their outcome assessments, the results of heterogeneity tests were *P*  = 0.382 and I^2^ = 0.0%, and *P*  = 0.992 and I^2^ = 0.0% respectively. Therefore, a fixed model was used to calculate the pooled HR, and the pooled HR for PFS was 1.20 (95% CI: 1.05–1.38, *P* = 0.007) (Fig. 2b) and 4.42 for RFS (95% CI: 2.14–9.15, *P* = 0.000) (Fig. 2b), indicating that high level of miR-210 expression was related to poor PFS, and even worse to RFS.

We also carried out subgroup analysis in breast cancer, renal cancer and sarcoma. Firstly, the HRs for OS (n = 5) and DFS (n = 4) in articles involving breast cancer were pooled. As an obvious heterogeneity (*P* = 0.000 and I^2^ = 82.3%) existed in these researches of OS, a random model was used to pool the HRs. The combined HR, 1.63 (95% CI: 0.47–5.67, *P* = 0.443) (Fig. S1) indicated that over-expressed miR-210 would not precisely predict poor OS for patients with breast cancer. We used a random model to calculate the combined HR for DFS, as there was heterogeneity (*P* = 0.061 and I^2^ = 59.3%). The pooled HR 2.03 (95% CI: 0.90–4.57, *P* = 0.088) (Fig. S2) showed miR-210 was a potential predictor of poor DFS in breast cancer. In the 3 articles about renal cancer using OS to assess outcome, the heterogeneity was significant (*P* = 0.016 and I^2^ = 76.0%), and the random model calculation produced the combined HR as 1.16 (95% CI: 0.27–4.94, *P* = 0.842) Fig. S1), implying the association between over-expressed miR-210 and poor OS was not significant in renal cancer. High heterogeneity (*P*  = 0.005 and I^2^ = 87.3%) was confirmed in 2 reports using OS as sarcoma outcome assessment value, and the combined HR reached 1.24 (95% CI: 0.20–7.89, *P* = 0.818) (Fig. S2) by calculating with a random model. Therefore, the over-expressed miR-210 may not exactly predict poor outcome of sarcoma patients.

### Heterogeneity Analysis Results

Obvious heterogeneity of subjects was found in 5 of the 8 analysis groups (OS for all, *P*<0.05, I^2^ = 72.0%; OS for breast cancer, *P* = 0.000 and I^2^ = 82.3%; OS for renal cancer, *P* = 0.016 and I^2^ = 76.0%; DFS for breast cancer, *P* = 0.061 and I^2^ = 59.3%, and OS for sarcoma, P = 0.005 and I^2^ = 87.3%). The most possible sources of heterogeneity were also analyzed by different methods. On one hand, since the heterogeneity of OS analysis group (14 studies) was obvious (*P*<0.05 and I^2^ = 72.0%), we divided the 14 studies into 3 cancer type-specific analysis groups (5 studies on breast cancer, 3 studies for renal cancer and 2 studies on sarcoma). The heterogeneity was still obvious in the 3sub-groups, so the cancer type could not solely explain the heterogeneity in OS analysis group. On the other hand, we conducted a meta regression to evaluate the potential factors responsible for the obvious heterogeneity. As a result, the publication year (*P* = 0.075), cut-off values (*P* = 0.228), patients origin (*P* = 0.252), risk evaluation method (*P* = 0.275), follow-up time (*P* = 0.280), cancer type (*P* = 0.453), sample size (*P* = 0.944) contributed to the heterogeneity to one degree or another. For the groups with less than 10 studies (meta regression is not proper to seek the sources of heterogeneity), we performed sensitivity analysis. In the OS analysis group for breast cancer, heterogeneity was significant (*P* = 0.000 and I^2^ = 82.3%). When Camps’ study was removed from analysis, the heterogeneity became insignificant (*P* = 0.228 and I^2^ = 30.7%). Therefore, we got a conclusion that Camps’ study was responsible for the heterogeneity. In another 2 analysis groups, Camps’, Wotschofsky’s study were responsible for the heterogeneity of DFS analysis group for breast cancer and OS analysis group for renal cancer respectively.

### Publication Bias

**Figure 3:**
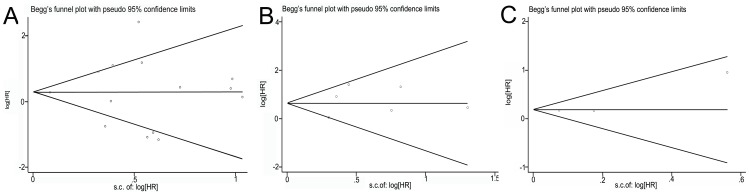
Funnel plots of studies included in the three meta-analysis: (A) overall survival, (B) disease-free survival and (C) progress-free survival.

Finally, the publication bias of included studies was evaluated by funnel plots and Egger’s tests. As shown in Fig. 3, the funnel plots were almost symmetric. In OS, DFS and PFS meta-analysis, the *P* values of Egger’s regression intercepts were 0.973, 0.578 and 0.378, respectively. Hence, there was no evidence for significant publication bias in our meta-analysis.

## Discussion

MiR-210 is an extensively studied hypoxia-related miRNA while hypoxia is an important pathophysiological process in solid cancer. Researches revealed that the expression of miR-210 was elevated in a variety of solid tumors, including breast cancer, non-small cell lung cancer, head and neck cancer, pancreatic cancer, oral tumors, hepatocellular cancer (HCC), adrenocortical carcinoma (ACC), colon cancer, ovarian cancer, glioblastoma, malignant melanoma and renal cell cancer. However, the down-regulation of miR-210 in human esophageal squamous cell carcinoma (ESCC) tissues and the derived cell lines was also reported [37], [38], [39], [40].

Transcriptionally regulated by hypoxia-induced factor (HIF), miR-210 controls the cellular response to hypoxia [5] and, in a way, helps cells adapt to hypoxia. Hypoxia-driven miR-210 directly targets E2F3 to inhibit cell proliferation in various cell lines including keratinocytes, ovarian cancer cells and human embryonic kidney cells [9], [41], [42]. E2F3 could promote cell proliferation by allowing cell cycle progression from G1 to S phase and the initiation of DNA replication [43], [44], [45]. Researches in cancer tissues and cells [41], [42] have proved miR-210 could inhibit cell proliferation via a fibroblast growth factor receptor (FGFR)-like 1 (FGFRL1) dependent mechanism since FGFRL1 promotes cell proliferation by facilitating cell cycle progression. Homeobox A1 (HOXA1) is also a target of miR-210, and over-expression of HOXA1 not only induced the activation of p44/42 MAP kinase to support cell proliferation but also reversed the inhibitory effect of miR-210 on cell growth. So, miR-210 could inhibit cell proliferation via targeting HOXA1 [46], [47]. However, over expressed miR-210 could inhibit cell death, promote cell survival by suppressing BCL2/adenovirus E1B 19 kDa protein-interacting protein 3 (BNIP3) in neural progenitor cells (NPC) [48]. Elevated miR-210 expression could also increase survival rates along with Akt and ERK activity in human mesenchymal stem cells (hMSCs) with hypoxia [49]. In addition, miR-210 is found to be one of the components responsible for radioresistance in human lung cancer cell lines H1975 [50]. What We should also noticed is that, by targeting MNT, a MYC antagonist, miR-210 promotes cell cycle progression in transformed cells such as colon cancer cells and cervical cancer cells [51]. This contradiction suggests a cell context-oriented effect of miR-210, and is supported by our data as the HRs varied with different cancer types.

MiR-210 depresses mitochondrial metabolism by targeting iron–sulfur cluster assembly homologue 1/2 (ISCU 1/2), which catalyzes the assembly of [4Fe-4S] and [2Fe-2S] Fe-S clusters to support mitochondrial functions [52], [53]. Besides, cytochrome oxidase assembly protein-10 (COX-10) and succinate dehydrogenase subunit D (SDHD) are all targets of miR-210 [53], [54]. So miR-210 may be a potent inhibitor in mitochondrial respiration by targeting TCA cycle and electron transport chain activity, and this down-tuning of glucose oxidation may decrease the harm of hypoxia, a condition inducing angiogenesis in solid tumors. On the other hand, over expressed miR-210 contributes to angiogenesis after cerebral ischemia by activating the Notch signaling pathway [55], while miR-210 inhibitor abrogates and 210 mimic recapitulates the pro-angiogenic effects by VEGF treatment in post-expansion CD34+ cells [56].

MiR-210 was recently reported to stall DNA repair by directly binding to the 3′ UTR of RAD52 [57], a protein that fixes DNA double-strand break, repairs single-stranded DNA gaps and facilitates RAD51-mediated strand invasion during homologous recombination [58], [59]. On the other hand, miR-210 promotes angiogenesis by inhibiting ephrin A3 (EFNA3) [60], [61], or directly targeting on protein-tyrosine phosphatase 1B (PTP1B) [61], [62]. Bianchi et al. reported a DNA-binding drug, mithramycin, promotes erythroid differentiation, induced the expression of miR-210 in erythroid progenitor cells [63]. Another report [64] showed miR-210 promotes bone morphogenic protein (BMP)-induced osteoblastic differentiation via targeting ACVR1B. In addition, miR-210 supports stem cell survival under hypoxia condition was demonstrated [65].

Up to now, the lab experimental results reveal that miR-210 may be essential for cancer cells to survival. In this meta-analysis, we got 8 pooled HRs from 1697 patients with 7 different cancers reported by 15 researches from 7 countries. In conclusion, the over-expression of miR-210 did predict poor survival of patients with cancers. Our analysis result was consistent with a previous meta-analysis [66] about miR-210 expression level and breast cancer patient survival. In the previous analysis, a pooled HR of the OS from only 4 records was 3.39 (95% CI: 2.04–5.63, *P*<0.05) for breast cancers, but the author wrongly assigned the HR for RFS to HR for OS in Toyama’s research [19].

Although the predictive effect of miR-210 was statistically proved, it should be carefully understood for following reasons. First, though the combined HR for OS was 1.62, considering its Pvalue 0.05, the predictive effect was not very strong. While the HRs of DFS, PFS, RFS, DFS for breast cancer and OS for sarcoma varied from 1.20 to 4.42 with all P values <0.015, but the numbers of researches for calculating HRs were all ≤5. Second, we only included articles in English, strictly, this might result in the miss of eligible researches published in other languages. Third, several HRs were calculated based on the data extracted from the survival curve, and this may bring errors although tiny. Fourth, we pooled HRs from different articles with different cut-off values due to methods limitations. We could not set up a baseline refering to miR-210 high expression either. The information of the included studies revealed that the heterogeneity could be attributed to the differences in the publication years, the types and stages of the cancers, the sample sizes, the cut-off values of miR-210, the durations of follow-up and the risk evaluation methods. We conducted sub-group analysis, meta regression and sensitive analysis to find out the possible sources of heterogeneity. So these factors and studies should be paid more attention to when the concerning conclusions were taken under consideration. Because differences might have a residual confounding effect within the relative studies, we attempted to minimize the effect by using a random effect model. Furthermore, although no significant publication bias was detected in the meta-analysis, the records size was not large enough to ensure the conclusion. Two researches [8], [33] about breast cancer collected their samples from patients after a period of treatment, 3 researches on HNSCC, breast cancer, bladder cancer [14], [17], [24] emphasized that their samples were got from untreated patients, while the rest 11 researches [19], [20], [22], [23], [29], [30], [31], [32], [34], [35], [36] did not exactly mention this issue. Theoretically, treatments may influence the expression of miR-210 in cancer samples, however, none of the researches referred to the treatment effect on HRs or miR-210 expression, and no conclusion could be drawn according to current data.

To date, miRNAs have been widely considered as oncogene/cancer suppressor, nevertheless, several concerns should be stressed. (i) Oncogenesis was a complicated process in our bodies involving numerous molecular pathways, and considering this, a set of miRNAs may more accurately represent cancer prognosis. (ii) Lacking of systemic standard of miRNAs or a standard reference miRNA made it difficult to explore their clinical application. For example, in the included researches, different cut-off values were used to stratify miR-210 expression levels. It is unclear whether each cancer type owns its specific cut-off value of individual miRNA, or shares the same cut-off value with others. (iii) Although several researches had reported the potential roles of miRNAs in chemo/radiation therapy [67], [68], [69], the efficiency and feasibility of miRNA therapies, such as interfering the expression of a specific miRNA or a set of them, and evaluating the side effect, still need to be investigated. (iv) Most of the included researches detected miRNA expression in cancer tissue samples, and that certainly matched the current clinical idea of golden standard for deterministic diagnosis. A simultaneous detection of miRNA in serum may give more information about host response and the prognosis although cancer tissue samples represent the condition of cancer tissue more appropriately than serum samples do.

## Conclusion

This meta-analysis summarized the global researches on the relationship of aberrant miR-210 expression and cancer patient survival, and clarified that over-expression of miR-210 in several cancers does predict poor survival of patients. Given the limitation of the current analysis, it should be cautious to appreciate the conclusion, and further clinical investigations are needed to testify the association between miR-210 and cancer prognosis as well as the efficiency of therapies.

## Supporting Information

Figure S1 **Subgroup analysis: Forrest plots of studies evaluating hazard ratios of high miR-210 expression as compared to low expression in subgroup analysis.** The random effect model is used to pool the HRs. As to overall survival for breast cancer, HR = 1.63 (95%CI: 0.47–5.67, *P* = 0.443), overall survival for renal cancer, HR = 1.16 (95%CI: 0.27–4.94, *P* = 0.842).
(TIF)

Figure S2 **Subgroup analysis: Forrest plots of studies evaluating hazard ratios of high miR-210 expression as compared to low expression in subgroup analysis.** The random effect model is used to pool the HRs. As to overall survival for sarcoma, HR = 1.24 (95%CI: 0.20–7.89, P = 0.818), disease-free survival for breast cancer, HR = 2.03 (95%CI: 0.90–4.57, P = 0.088).
(TIF)

Checklist S1 **Preferred Reporting Items for Systematic Reviews and Meta-Analyses (PRISMA) 2009 Checklist.**
(DOC)

Checklist S2 **Meta-analysis of Observational Studies in Epidemiology group (MOOSE) Checklist.**
(DOC)
