# Supplementary figures and images for: Elevated Expression of miR-210 Predicts Poor Survival of Cancer Patients: A Systematic Review and Meta-Analysis
Source: PLoS One. 2014 Feb 20;9(2):e89223. doi: 10.1371/journal.pone.0089223 (PMC3930667; doi:10.1371/journal.pone.0089223)

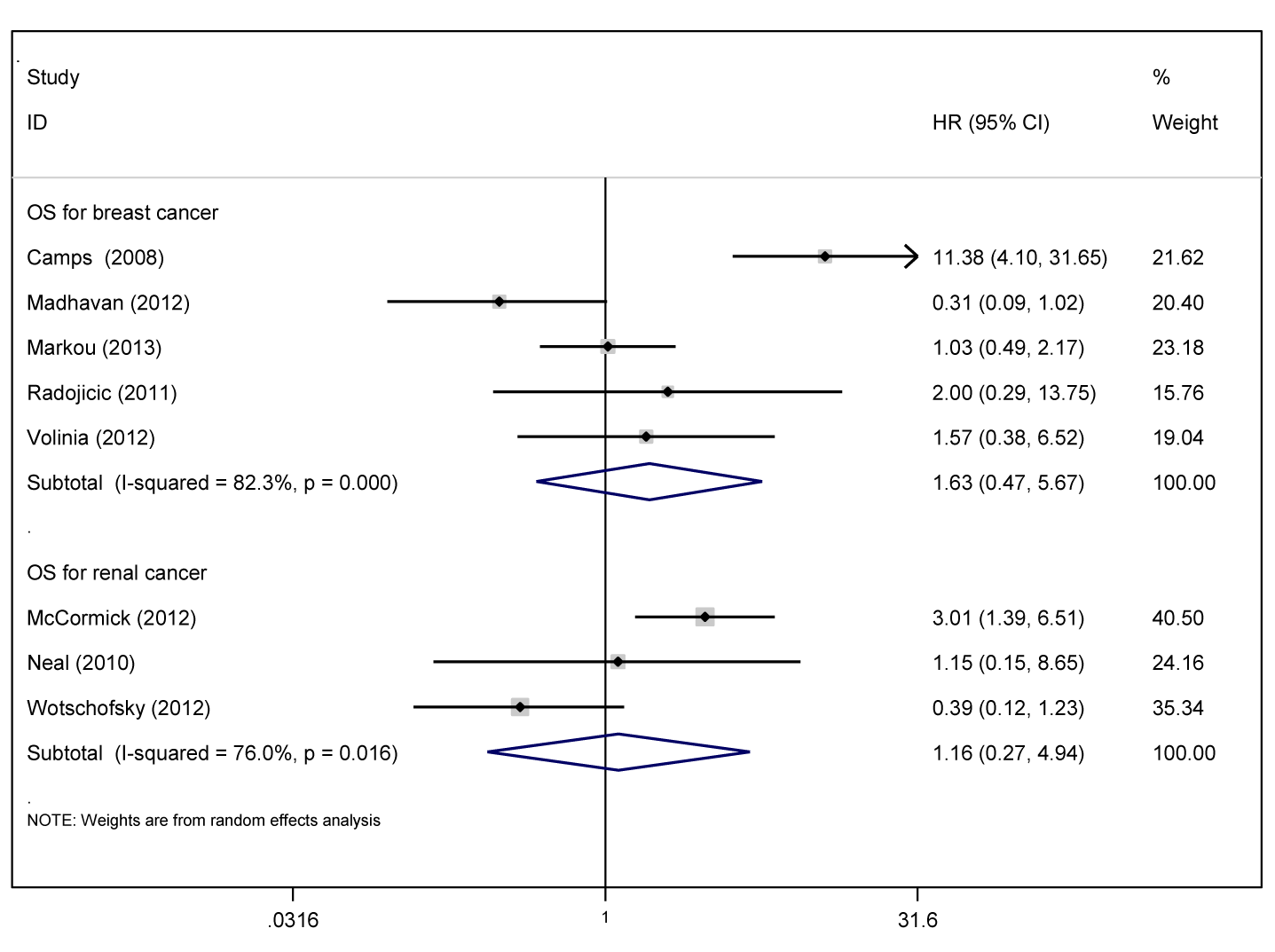

Supplement: Figure S1 — Subgroup analysis: Forrest plots of studies evaluating hazard ratios of high miR-210 expression as compared to low expression in subgroup analysis. The random effect model is used to pool the HRs. As to overall survival for breast cancer, HR = 1.63 (95%CI: 0.47–5.67, P = 0.443), overall survival for renal cancer, HR = 1.16 (95%CI: 0.27–4.94, P = 0.842). (TIF) [file pone.0089223.s001.tif]

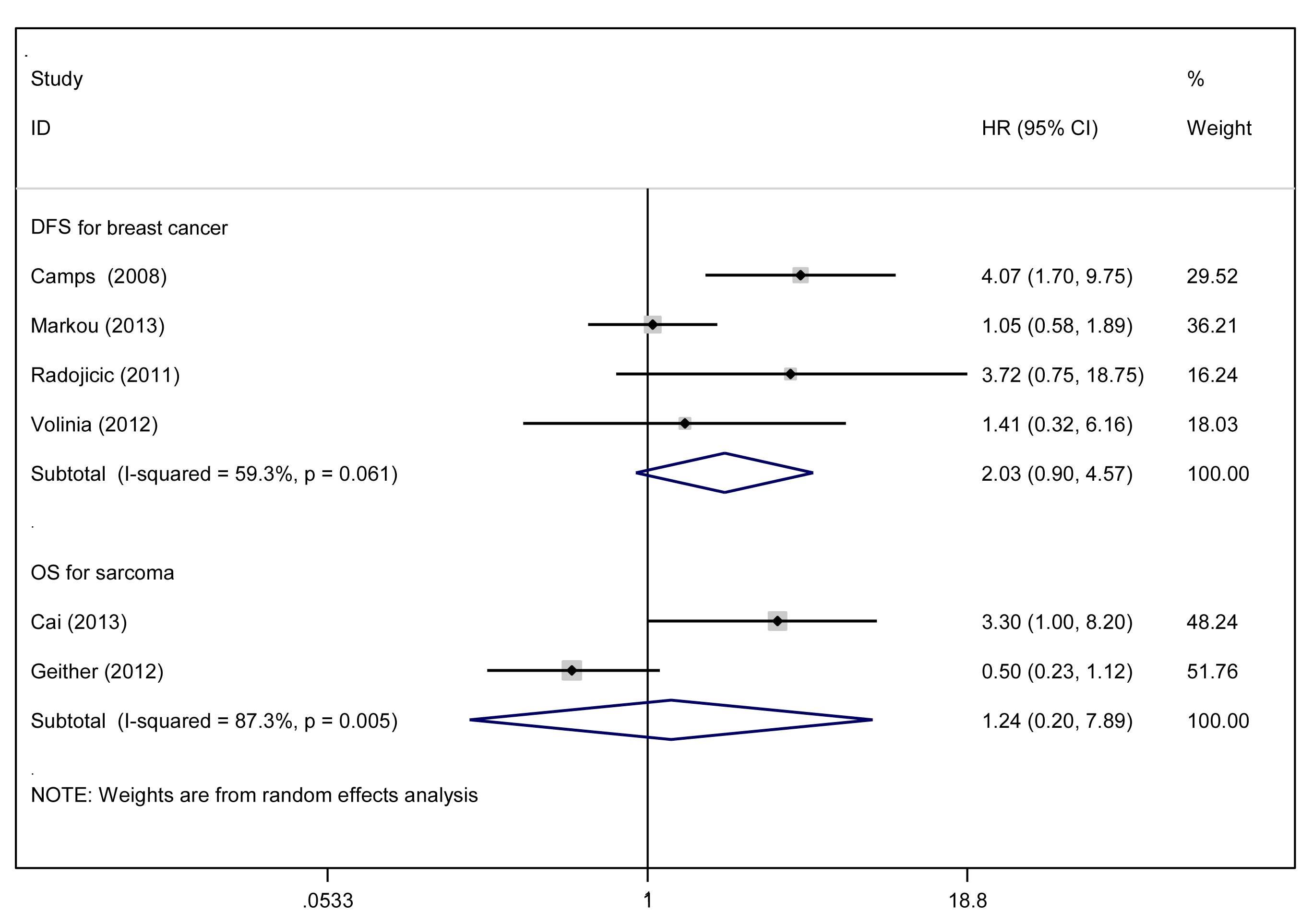

Supplement: Figure S2 — Subgroup analysis: Forrest plots of studies evaluating hazard ratios of high miR-210 expression as compared to low expression in subgroup analysis. The random effect model is used to pool the HRs. As to overall survival for sarcoma, HR = 1.24 (95%CI: 0.20–7.89, P = 0.818), disease-free survival for breast cancer, HR = 2.03 (95%CI: 0.90–4.57, P = 0.088). (TIF) [file pone.0089223.s002.tif]
