# Supplementary material for: Elevated Expression of miR-210 Predicts Poor Survival of Cancer Patients: A Systematic Review and Meta-Analysis
Source: PLoS One. 2014 Feb 20;9(2):e89223. doi: 10.1371/journal.pone.0089223 (PMC3930667; doi:10.1371/journal.pone.0089223)
Supplement: Checklist S2 — Meta-analysis of Observational Studies in Epidemiology group (MOOSE) Checklist. (DOC) [file pone.0089223.s004.doc]

**Table S2**. MOOSE checklist

| **Reporting of background should include** | |
| --- | --- |
| Problem definition | Background |
| Hypothesis statement | Background |
| Description of study outcome(s) | OS, DFS, PFS, RFS |
| Type of exposure or intervention used | cancer |
| Type of study designs used | Systematic reviews and meta-analysis |
| Study population | global |
| **Reporting of search strategy should include** | |
| Qualifications of searchers (eg, librarians and investigators) | Stated in methods |
| Search strategy, including time period included in the synthesis and keywords | Methods |
| Effort to include all available studies, including contact with authors | We contact authors and searched reference lists and citations |
| Databases and registries searched | Methods |
| Search software used, name and version, including special features used (eg, explosion) | 360 secured browser 6.2 |
| Use of hand searching (eg, reference lists of obtained articles) | Methods |
| List of citations located and those excluded, including justification | Flow diagram in Figure 1. |
| Method of addressing articles published in languages other than English | Method |
| Method of handling abstracts and unpublished studies | Method |
| Description of any contact with authors | Method |
| **Reporting of methods should include** | |
| Description of relevance or appropriateness of studies assembled for assessing the hypothesis to be tested | Method |
| Rationale for the selection and coding of data (eg, sound clinical principles or convenience) | Methods |
| Documentation of how data were classified and coded (eg, multiple raters, blinding, and interrater reliability) | Methods |
| Assessment of confounding (eg, comparability of cases and controls in studies where appropriate) | Methods |
| Assessment of study quality, including blinding of quality assessors; stratification or regression on possible predictors of study results | Methods |
| Assessment of heterogeneity | Methods |
| Description of statistical methods (eg, complete description of fixed or random effects models, justification of whether the chosen models account for predictors of study results, dose-response models, or cumulative meta-analysis) in sufficient detail to be replicated | Methods |
| Provision of appropriate tables and graphics | Methods |
| **Reporting of results should include** | |
| Graphic summarizing individual study estimates and overall estimate | Figure 2, 3, S1, S2 |
| Table giving descriptive information for each study included | Table 1, 2, 3 |
| Results of sensitivity testing (eg, subgroup analysis) | Figure S1, S2 |
| Indication of statistical uncertainty of findings | Discussion |
| **Reporting of discussion should include** | |
| Quantitative assessment of bias (eg, publication bias) | Result |
| Justification for exclusion (eg, exclusion of non–English-language citations) | Discussion |
| Assessment of quality of included studies | Results and discussion |
| **Reporting of conclusions should include** | |
| Consideration of alternative explanations for observed results | Discussion |
| Generalisation of the conclusions (ie, appropriate for the data presented and within the domain of the literature review) | Discussion |
| Guidelines for future research | Discussion |
| Disclosure of funding source | submission system |
